# Optimal ICSI timing on immature oocytes for low prognosis patients under the POSEIDON classification

**DOI:** 10.1186/s12884-024-06577-x

**Published:** 2024-06-06

**Authors:** Cheng Shi, Ping Wang, Rong Liang, Min Fu, Sheng Nan Duan, Huan Shen, Mei Yang, Xi Chen

**Affiliations:** 1 Reproductive Medical Center, Department of Obstetrics and Gynecology, Peking University People’s Hospital, Peking University, Beijing, 100044 China; 2 Beijing Qiaozhao Xinye Biology Science and Technology Company Co., Ltd, Beijing, China

**Keywords:** ICSI timing, Immature oocytes, POSEIDON patients, GEE, Mitochondria distribution

## Abstract

**Background:**

The optimal timing of performing ICSI on immature oocytes for POSEIDON patients is still unknown to get better early embryonic development outcomes. The purpose of this study was to implore the most appropriate time to carry out ICSI on in vitro maturation GV and MI oocytes for POSEIDON patients.

**Methods:**

Two hundred thirty-nine immature oocytes from 163 POSEIDON patients were prospectively performed ICSI at different timings: P-ICSI (ICSI was performed on in vitro matured oocytes 4–6 h after the first polar body extrusion, *N* = 81), R-ICSI (ICSI was performed on in vitro matured oocytes less than 4 h after the first polar body extrusion, *N* = 80), and E-ICSI (ICSI was performed on in vitro matured oocytes the next day after oocytes retrieval, *N* = 78). Fertilization and embryonic development outcomes were collected and statistically analyzed. Mitochondria distribution of cytoplasm of in vitro matured oocytes with different time cultures after the first polar body (PB1) extrusion was stained.

**Results:**

Compared to the E-ICSI group, more day 3 embryos from P-ICSI became blastocysts after sequential culture though without statistical significance (OR = 3.71, 95% CI: 0.94—14.63, *P* = 0.061). Compared to the E-ICSI group, more embryos from both P-ICSI and R-ICSI groups were clinically used with statistical significance (OR = 5.67, 95% CI: 2.24—14.35, *P* = 0.000 for P-ICSI embryos; OR = 3.23, 95% CI: 1.23—8.45, *P* = 0.017 for R-ICSI embryos). Compared to the E-ICSI group, transferred embryos from P-ICSI and R-ICSI had a higher implantation rate though without statistical significance (35.3% for P-ICSI embryos; 9.1% or R-ICSI embryos and 0% for E-ICSI embryos, *P* = 0.050). Among the three group, there were most healthy babies delivered from the P-ICSI group (5, 1 and 0 for P-ICSI, R-ICSI and E-ICSI respectively). The mitochondria in the cytoplasm of in vitro matured oocytes with a less than 4 h and 4–6 h culture after PB1 extrusion presented semiperipheral and diffused distribution patterns, respectively.

**Conclusions:**

Our results revealed P-ICSI (ICSI was performed on in vitro matured oocytes 4–6 h after the first polar body extrusion) provided the most efficient method to utilize the immaturation oocytes basing on embryos utilization and live birth outcome for low prognosis patients under the POSEIDON classification. The mitochondria distribution of the in vitro matured oocytes’ cytoplasm from P-ICSI varied that from R-ICSI.

## Background

In China, more than 30% of women who undergo In vitro fertilization-embryo transfer (IVF-ET) treatment may be classified as having a low prognosis under the new, more detailed Patient-Oriented Strategies Encompassing Individualized Oocyte Number (POSEIDON) criteria [1]. However, according to the latest reports, the cumulative delivery rate (CDR) was lower in the POSEIDON patients than in the non-POSEIDON patients [2, 3]. Thus, managing low prognosis under POSEIDON criteria patients is challenging in reproductive medicine. Notably, there were about 11% metaphase I (MI) and 4% germinal vesicle (GV) immature oocytes in routine IVF-ET cycles, including POSEIDON patients [4]. For normal responders with an adequate ovarian reserve, most IVF centers do not use such immature oocytes and discard them. In a single stimulation cycle, an increased oocyte yield may increase the number of embryos potentially obtained, thereby enhancing the possibility of a live birth delivery after transfer [5]. Therefore, for low prognosis patients, each immature oocyte has a clinical value if it can be fertilized and developed normally [6].

Most immature oocytes could spontaneously extrude the first polar body (PB1) when maintained in a culture medium. However, for these in vitro maturation (IVM) MI and GV oocytes, there is still no consensus regarding the optimal intracytoplasmic sperm injection (ICSI) timing after PB1 extrusion, which has been reported to be critical for these oocytes’ embryonic development outcome [7, 8]. In most cases, ICSI was performed on these in vitro maturation MI and GV oocytes at the same time as in vivo-maturation metaphase II (MII) oocytes, and the interval between PB1 extrusion and ICSI performing was less than 4 h. Considering the cytoplasm also needs a longer time to mature after nuclear maturation for in vitro maturation of oocytes, a 4–6 h interval was recommended in a previous study [9]. When PB1 is extruded outside working hours, ICSI can only be performed the next day of oocyte retrieval, which had been reported to result in a decline in fertilization and early embryo developmental outcome [10]. It is still unknown what the optimal ICSI timing is for the immature oocytes from low prognosis patients under POSEIDON criteria. Therefore, the primary purpose of this study was to implore the most appropriate time to carry out ICSI on in vitro maturation GV and MI oocytes based on better early embryonic development outcomes for low prognosis patients under the POSEIDON classification.

## Materials and methods

### Patients selection and study design

This study was a prospective, observational study. The study population comprised infertility patients who intended to receive ICSI treatment in our reproductive center from April 2019 to January 2022. Patients who retrieved no more than 9 oocytes and had at least one immature oocyte were included. The patients whose husbands were diagnosed with azoospermia and severe oligoasthenoteratozoospermia (OAT) and whose immature oocytes could not extrude PB1 within 24 h of in vitro culture were excluded. This study was approved by the Ethics Review Board of the Peking University People’s Hospital (2018PHB186-01). Furthermore, each participant was informed in detail about the study’s procedures and risks, and the informed consent was obtained.

### Ovarian stimulation

Each patient accepted a controlled ovarian stimulation (COS) regimen such as long, ultralong, and micro-stimulation protocol according to their ovarian function and responses during previous IVF cycles or ovulation induction. The diameter of follicles was monitored every 2–3 days, and ovulation was induced with human chorionic gonadotropin (hCG; Choragon, Ferring, Switzerland, 5000–10,000 IU) alone or in combination with triptorelin acetate (Ferring, 0.2 mg) when at least two leading follicles grew up to 18 mm.

### Oocytes retrieval and cumulus cells denudation

Thirty-six hours after hCG administration, transvaginal ultrasound-guided oocytes retrieval was performed. The retrieved oocyte-corona radiata-cumulus oophorus complexes (OCCCs) were incubated in fertilization medium in a 60 mm IVF dish (353653; Falcon, Franklin Lakes, NJ, USA). The cumulus cells of the patients’ OCCCs were removed 2 h after retrieval. The OCCCs were briefly exposed to 75 IU/mL hyaluronidase (SAGE) and then mechanically denuded by a set of pipettes until there left few cumulus cells. The denuded oocytes were evaluated under an inverted microscope to assess the nuclear maturation stages (GV, MI, and MII). Furthermore, the oocytes at the MII stage were processed according to clinical standard operating procedure and the oocytes at MI and GV stage were selected for the ongoing study.

### Immature oocytes in vitro culture, grouping, and ICSI performing

Following evaluation, the MI and GV stage oocytes were then separately cultured in different microdroplets with the same cleavage medium (30 μl; G1-PLUS, Vitrolife, Sweden) and reevaluated every 1 h until workover of the oocytes retrieval day. If the PB1 of the immature oocyte was extruded before routine ICSI performance timing (40 h after hCG administration), the oocyte was assigned to the routine-ICSI (R-ICSI) or precise-ICSI (P-ICSI) group according to a randomized format. Oocytes in the R-ICSI group were cultured in the cleavage medium for less than 4 h after PB1 extrusion. Then they were performed ICSI simultaneously with in-vivo matured MII oocytes ( 40 h after hCG administration). In comparison, oocytes in the P-ICSI group were cultured in the cleavage medium for 4–6 h after PB1 extrusion and then were performed ICSI. If the PB1 was extruded outside our working hours but within 24 h, the oocytes were performed ICSI the next day and were assigned to the extended-ICSI (E-ICSI) group.

### Embryo culture, evaluation, processing, and outcomes calculation

After ICSI performing, the inseminated IVM oocytes were immediately transferred to the culture dish in cleavage medium and put into a COOK incubator with a mixed gas of 5% O_2_, 6% CO_2_, and 89% N_2_ for embryo culture. Additionally, the pronuclei were examined under a microscope at 16–18 h after ICSI. Normally fertilized zygotes (with two pronuclei) were placed individually in a single microdrop for the ongoing culture. Moreso, morphological evaluation of day 3 embryos was performed using the modified Pruissant scoring criteria as mentioned before [11]. Day 3 embryos were classified into four grades according to embryo cleavage speed, blastomeres' uniformity, and fragmentation ratio. Embryos with grade I were evaluated as morphological good embryos (MGE), while embryos with grade I or II were evaluated as utilizable. Embryos with grade III or IV were cultured in blastocyst medium (30 μl; G2-PLUS, Vitrolife, Sweden) for another 2 to 4 days. Day 5, 6, or 7 blastocysts were classified into three grades according to blastocele's expansion state, inner cell mass quality, and trophectoderm cells. Good or median blastocysts (at least trophectoderm or inner cell mass score ‘B’or ‘A’) were evaluated as utilizable embryos. Notably, these utilizable embryos from immature oocytes were not considered with priority for clinical transfer. Only in case of no better embryos originating from MII stage oocytes, one or two embryos originating from the immature oocytes,were transferred in a fresh or thawing cycle. Fourteen days after embryo transfer, hCG in blood was tested to determine whether the patient was clinically pregnant or not, and an ultrasound was performed on 28 days to determine the number of fetal sacs.

The major outcomes were calculated as follows:

- 2PN zygotes rate = number of 2PN zygotes/number of ICSI oocytes.
- 7–10 cell day 3 embryos rate = number of embryos with 7–10 blastomeres on day 3/number of embryos cleaved and cultivated until day 3.
- MGE rate = number of MGE on day 3/number of embryos cleaved and cultivated until day 3.
- Blastocyst formation rate = number of blastocysts/ number of embryos performing blastocyst cultivation.
- Utilizable embryos rate = number of embryos utilized clinically (transfer and freezing)/number of cleaved embryos.
- Implantation rate = number of successfully implanted embryos/number of transferred embryos.

### Statistical analysis

Since not all the demographic and treatment cycle characteristics of participants from the three groups were normally distributed, these data were analyzed by one-way analysis of variance (ANOVA) or the Kruskal–Wallis H test. Continuous variables were presented as mean ± standard or median (Q1, Q3). Categorical variables were expressed in frequency and percentage, and a Chi-square test was performed. Uni-variate analysis was employed to test the impact of each variable on the major outcomes. To find the real effect of ICSI timing on fertilization and embryonic development outcomes, multivariable logistic regression analysis was employed by adjusting clinical and significant co-variables. Since each participant may have several oocytes, which may be assigned to different ICSI timing groups, a generalized estimate equation (GEE) model based on the participants’ unique medical record number was used both in the univariate and multivariable logistic regression analysis to avoid the repeated measurement effect of participant characteristics. Furthermore, all data were analyzed by statistical product and service solutions (SPSS) 23.0 (IBM, NYU). *P*-values less than 0.05 (two-sided) were considered statistically significant.

### Mitochondria distribution staining on cytoplasm of IVM oocytes with a less than 4 h and 4–6 h culture after PB1 extrusion

Tetramethylrhodaminemethyl ester (TMRM) fluorescence was detected using a laser scanning confocal microscopy (Leica TCS SP8 STED; Mannheim, Germany) to indicate the mitochondria distribution of the cytoplasm. Briefly, approximately 3 and 3 oocytes, respectively, were incubated at less than 4 h and 4–6 h culture after PB1 extrusion with 100 nM TMRM (Invitrogen) at 37 ℃ for 30 min. The fluorescence of TMRM was detected by Leica TCS SP8 STED confocal microscopy with an excitation wavelength of 525 nm.

## Results

### Baseline characteristics of participants

**Fig. 1:**
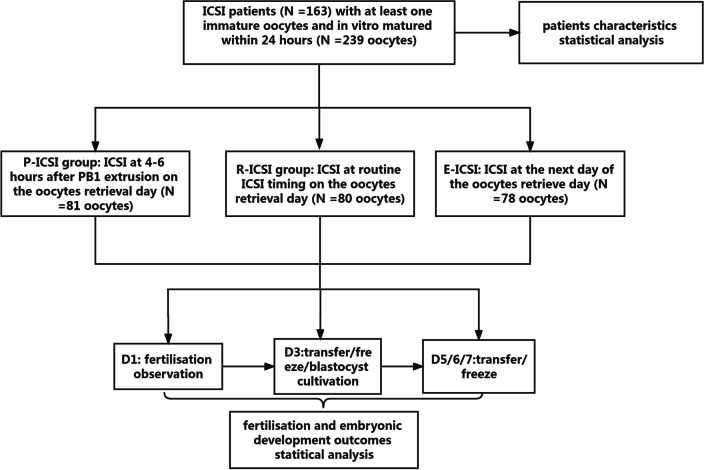
Flow chart of study design and analysis

A total of 163 participants were enrolled, and 239 immature oocytes were successfully grouped according to ICSI timing (Fig. 1). Participants’ demographic and treatment cycle characteristics are shown in Table 1. Among P-ICSI, R-ICSI, and E-ICSI groups, no significant differences were found in maternal age, cycle number, the level of anti-Müllerian hormone (AMH) and basic follicle stimulation hormone (FSH), infertility duration, number of retrieved oocytes and total gonadotropin dosages.

**Table 1:** Demographic and treatment cycle characteristics of all participants (*N* = 163)

Characteristics	P-ICSI^a^ (*N* = 60)	R-ICSI^b^ (*N* = 60)	E-ICSI^c^ (*N* = 43)	*P*-value
Maternal age (years)	35.65 ± 4.09	35.95 ± 4.20	35.65 ± 5.12	0.917*
Cycle number (number, median, Q1-Q3)	2 (1–3)	2 (1–2.75)	1 (1–2)	0.242^#^
AMH (ng/ml,median, Q1-Q3)	1.31(0.75–2.11)	1.56(0.77–2.29)	1.78(0.86–3.13)	0.441^#^
Infertility duration (years, median, Q1-Q3)	3 (2–5)	3 (2–6)	3 (2–5)	0.644^#^
Basel FSH (mIU/ml,median, Q1-Q3)	8.63(6.76–10.61)	8.05(6.76–10.17)	7.61(6.57–10.49)	0.247^#^
Retrieved oocytes (number, median, Q1-Q3)	5 (3–6)	5 (3–7)	5 (3–6)	0.572^#^
Total gonodotropin dosages (IU)	2582.10 ± 1001.29	2685.22 ± 1144.51	2580.81 ± 1090.99	0.840*
Average gonodotropin dosages (IU)	291.58 ± 79.27	285.60 ± 84.06	299.28 ± 88.00	0.717*

*AMH* anti-Müllerian hormone, *FSH* Follicle stimulation hormone

^*^Analyzed by one-way analysis of variance (ANOVA)

^#^Analyzed by Kruskal–Wallis H test

^a^ICSI was performed on oocytes 4–6 h after the first polar body extrusion

^b^ICSI was performed on oocytes at 40 h after hCG administration, less than 4 h after the first polar body extrusion

^c^ICSI was performed on oocytes the next day after oocytes retrieval

### Fertilization, cleaving speed, quality, blastocyst formation, utilizable embryo rate, and implanting results of IVM embryos resulting from different ICSI timing groups

A total of 239 immature oocytes extruded the PB1. They were assigned into three ICSI timing groups according to the time of performing ICSI: 81 oocytes in the P-ICSI group, 80 in the R-ICSI group, and 78 in the E-ICSI group (Fig. 1). The 2PN zygotes rate after ICSI performing were respectively 72.8%, 66.3%, and 67.9% in P-ICSI, R-ICSI, and E-ICSI groups. Moreso, 96.7%, 96.5%, and 96.4% of the 2PN zygotes in the P-ICSI, R-ICSI, and E-ICSI groups were cleaved and cultivated until day 3. The proportion of embryos with 7–10 cells on day 3 was 35.6%, 28.3%, and 18.9%, and the MGE rate on day 3 were 6.8%, 7.5%, and 1.9%, respectively, in the P-ICSI, R-ICSI, and E-ICSI groups. Furthermore, 32, 29, and 42-day 3 cleaved embryos from P-ICSI, R-ICSI, and E-ICSI groups were cultivated in blastocyst medium, and the blastocyst formation rate were 34.4%, 13.8%, and 11.9%, respectively (Table 2).

**Table 2:** Fertilization, cleaving speed, quality of day 3 embryos, blastocyst formation, and utilizable embryo rate of IVM embryos resulting from different ICSI timing groups (*N* = 239)

Characteristics	ICSI timing group		
	P-ICSI	R-ICSI	E-ICSI
No. of oocytes	81	80	78
2PN zygote rate, n (%)^a^	59 (72.8)	53 (66.3)	53 (67.9)
7–10 cell embryo rate, n (%)^b^	21 (35.6)	15 (28.3)	10 (18.9)
MGE rate, n (%)^c^	4 (6.8)	4 (7.5)	1 (1.9)
Blastocyst formation rate, n (%)^d^	11 (34.4)	4 (13.8)	5 (11.9)
Utilizable embryo rate, n (%)^e^	39 (65)	28 (50)	16(30.2)

*MGE* morphological good embryos:7–10 even cells with less than 15% fragmentation, *IVM* in vitro maturation

^a^number of 2PN zygotes/number of ICSI oocytes

^b^number of embryos with 7–10 blastomeres on day 3/number of embryos cleaved and cultivated until day 3

^c^number of MGE on day 3/number of embryos cleaved and cultivated until day 3

^d^number of blastocysts/ number of embryos performing blastocyst cultivation

^e^number of embryos utilized clinically (transfer and freezing)/number of cleaved embryos

Day 3 embryos evaluated grade I or II, and good or moderate blastocysts were clinically utilizable embryos, which were frozen or transferred. Additionally, 65%, 50%, and 30.2% of embryos, respectively, from P-ICSI, R-ICSI, and E-ICSI groups were utilized (Table 2). Moreso, 17, 11, and 10 embryos from P-ICSI, R-ICSI, and E-ICSI groups were transferred, and 6, 1, and 0 embryos were implanted successfully. The implantation rate of embryos from the P-ICSI group was higher than that from the E-ICSI group but without significance (*P* = 0.050;) (Table 3). Finally, 5 and 1 healthy babies had been delivered from P-ICSI and R-ICSI group seperately.

**Table 3:** Implantation results of transferred embryos from different ICSI timing groups (*N* = 38)

Characteristics	P-ICSI	R-ICSI	E-ICSI	*P*-value*
No. of transferred embryos	17	11	10	-
Implantation rate, n (%)^a^	6 (35.3)	1 (9.1)	0 (0)	0.050
Live birth, n	5	1	0	

^a^Number of successfully implanted embryos/number of transferred embryos

^*^Analyzed by Chi-square test

### Impact of each variable on fertilization, cleaving speed, quality, blastocyst formation, and the clinical result of IVM embryos based on the GEE model

For the outcome of fertilization (recorded as 'whether the zygote was 2PN or not after ICSI'; a binary variable), infertility duration had significant power. Along with the increased infertility duration of female participants, fewer oocytes became 2PN zygotes after ICSI (OR = 0.92, 95% CI: 0.86—0.97, *P* = 0.004; analyzed by univariable logistic regression-GEE model). For the outcome of cleaving speed (recorded as 'whether the embryo cleaved to 7–10 cells on Day 3 or not'; a binary variable), comparing to E-ICSI oocytes, more P-ICSI zygotes cleaved to 7–10 cells on Day 3 but without significance (OR = 2.16, 95% CI: 0.87—5.37, *P* = 0.099; analyzed by univariable logistic regression-GEE model). Maternal age had significant power for the embryo quality outcome (recorded as 'whether the embryo was an MGE on Day 3 or not’; a binary variable). Along with female participants' increased age, fewer embryos were evaluated MGE on day 3 (OR = 0.88, 95% CI: 0.79—0.98, *P* = 0.015; analyzed by univariable logistic regression-GEE model). For the outcome of the blastocyst formation (recorded as 'whether the cultivated day 3 embryo became a blastocyst on day 5/6/7 or not'; a binary variable), compared to E-ICSI oocytes, more P-ICSI embryos became blastocysts after cultivation but without significance (OR = 3.62, 95% CI: 0.93—14.06, *P* = 0.063; analyzed by univariable logistic regression-GEE model). For the outcome of utilizable embryos (recorded as 'whether the embryo was clinically used or not'; a binary variable), maternal age, number of retrieved oocytes, and ICSI timing had significant power. Specifically, more embryos were frozen or transferred along with the increased age and cycle number of female participants (OR = 1.10, 95% CI: 1.02—1.19, *P* = 0.011 for maternal age; analyzed by univariable logistic regression-GEE model). In contrast, fewer embryos were frozen or transferred along with the increased number of retrieved oocytes (OR = 0.70, 95% CI: 0.58—0.85, P = 0.000; analyzed by univariable logistic regression-GEE model). Compared to E-ICSI embryos, more P-ICSI and R-ICSI embryos were frozen or transferred (OR = 4.44, 95% CI: 1.82—10.80, *P* = 0.001 for P-ICSI embryos; OR = 2.61, 95% CI: 1.02—6.67, *P* = 0.046 for R-ICSI embryos; analyzed by univariable logistic regression-GEE model) (Table 4).

**Table 4:** Uni-variate analysis for fertilization, cleaving speed, quality of Day 3 embryos, blastocyst formation, and utilizable embryo of IVM embryos

	7–10 cell embryo		2PN zygote		MGE		Blastocyst formation		Utilizable embryo	
Covariates	EXP(OR) 95%CI	P-value	EXP(OR) 95%CI	P-value	EXP(OR) 95%CI	P-value	EXP(OR) 95%CI	P-value	EXP(OR) 95%CI	P-value*
ICSI-timing										
P-ICSI	2.16 (0.87,5.37)	0.099	1.06 (0.59,1.92)	0.845	4.48 (0.28,72.55)	0.292	3.62 (0.93,14.06)	0.063	4.44 (1.82,10.80)	*0.001*
R-ICSI	1.65 (0.62,4.43)	0.316	0.87 (0.45,1.67)	0.669	6.13 (0.34,109.32)	0.218	1.13 (0.20,6.40)	0.894	2.61 (1.02,6.67)	*0.046*
E-ICSI	Refrence		Refrence		Refrence		Refrence		Refrence	
Maternal age	0.98 (0.90,1.07)	0.63	0.95 (0.89,1.00)	0.071	0.88 (0.79,0.98)	*0.015*	1.02 (0.91,1.14)	0.769	1.10 (1.02,1.19)	*0.011*
Cycle number	1.07 (0.87,1.30)	0.526	1.14 (0.95,1.37)	0.17	0.57 (0.32,1.01)	*0.054*	1.05 (0.76,1.46)	0.759	1.21 (0.98,1.50)	0.073
AMH	0.91 (0.69,1.19)	0.477	1.02 (0.86,1.21)	0.83	1.18 (0.90,1.55)	0.226	1.00 (0.71,1.42)	0.989	0.85 (0.68,1.06)	0.148
Infertility duration	0.93 (0.87,1.06)	0.276	0.92 (0.86,0.97)	*0.004*	0.76 (0.49,1.16)	0.203	0.94 (0.76,1.16)	0.569	0.94 (0.83,1.05)	0.262
Basel FSH	1.06 (0.97,1.16)	0.185	0.98 (0.93,1.02)	0.244	1.03 (0.91,1.16)	0.618	0.97 (0.86,1.11)	0.664	1.04 (0.95,1.13)	0.407
Retrieved oocytes	0.91 (0.75,1.10)	0.336	1.00 (0.88,1.12)	0.928	1.09 (0.75,1.58)	0.647	0.96 (0.70,1.31)	0.792	0.70 (0.58,0.85)	*0.000*
Total gonodotropin dosages	1.00 (1.00,1.00)	0.376	1.00 (1.00,1.00)	0.303	1.00 (1.00,1.00)	0.519	1.00 (1.00,1.00)	0.616	1.00 (1.00,1.00)	0.528

*AMH* anti-Müllerian hormone, *FSH* Follicle stimulation hormone, *MGE* morphological good embryo:7–10 even cells with less than 15% fragmentation

^*^Analysis were done by univariable logistic regression-GEE model (Generalized estimate equation) model, subject ID = patient unique medical record number (independence)

The italic *P*-value indicates a significant difference

### Effects of ICSI timing on cleaving speed of day 3 embryos, blastocyst formation, and utilizable embryo in multivariable logistic regression-GEE models

For the cleaving speed of day 3 embryos, maternal age, AMH, and infertility duration were adjusted in the multivariable logistic regression-GEE model, and ICSI timing had no significant effect (OR = 2.06, 95% CI: 0.80—5.34, *P* = 0.136 for P-ICSI embryos; OR = 1.70, 95% CI: 0.61—4.72, *P* = 0.31 for R-ICSI embryos; analyzed by multivariable logistic regression-GEE model) (Table 5).

**Table 5:** Effects of ICSI timing on the cleaving speed of day 3 embryos, blastocyst formation, and utilizable embryo in multivariable logistic regression-GEE models

Variable	7–10 cell embryo^a^		Blastocyst formation^b^		Utilizable embryo^c^	
	OR (95%CI)	*P-*value	OR (95%CI)	*P-*value	OR (95%CI)	*P-*value*
ICSI-timing						
P-ICSI	2.06 (0.80,5.34)	0.136	3.71 (0.94,14.63)	0.061	5.67 (2.24,14.35)	*0.000*
R-ICSI	1.70 (0.61,4.72)	0.310	1.14 (0.17,7.57)	0.895	3.23 (1.23,8.45)	*0.017*
E-ICSI	Refrence		Refrence		Refrence	

^a^Adjusted for maternal age, AMH, and infertility duration

^b^Adjusted for maternal age, AMH, and infertility duration

^c^Adjusted for maternal age, cycle number, and retrieved oocytes

^*^Analysis were done by multivariable logistic regression-GEE model (Generalized estimate equation) model, subject ID = patient unique medical record number (independence)

The italic *P*-value indicates a significant difference

For blastocyst formation outcome, maternal age, AMH, and infertility duration were adjusted in the multivariable logistic regression-GEE model. Compared to E-ICSI embryos, more P-ICSI embryos became blastocysts after cultivation though without significance (OR = 3.71, 95% CI: 0.94—14.63, *P* = 0.061; analyzed by multivariable logistic regression-GEE model) (Table 5).

For utilizable embryos outcome, maternal age, cycle number, and retrieved oocytes were adjusted in the multivariable logistic regression-GEE models, and ICSI timing still had a significant effect on utilizable embryos. Compared to E-ICSI oocytes, more P-ICSI and R-ICSI oocytes were frozen or transferred (OR = 5.67, 95% CI: 2.24—14.35, *P* = 0.000 for P-ICSI embryos; OR = 3.23, 95% CI: 1.23—8.45, *P* = 0.017 for R-ICSI embryos; analyzed by multivariable logistic regression-GEE model) (Table 5).

### Mitochondria distribution of the cytoplasm of in vitro maturation oocytes with a less than 4 h and 4–6 h cultivation after PB1 extrusion

**Fig. 2:**
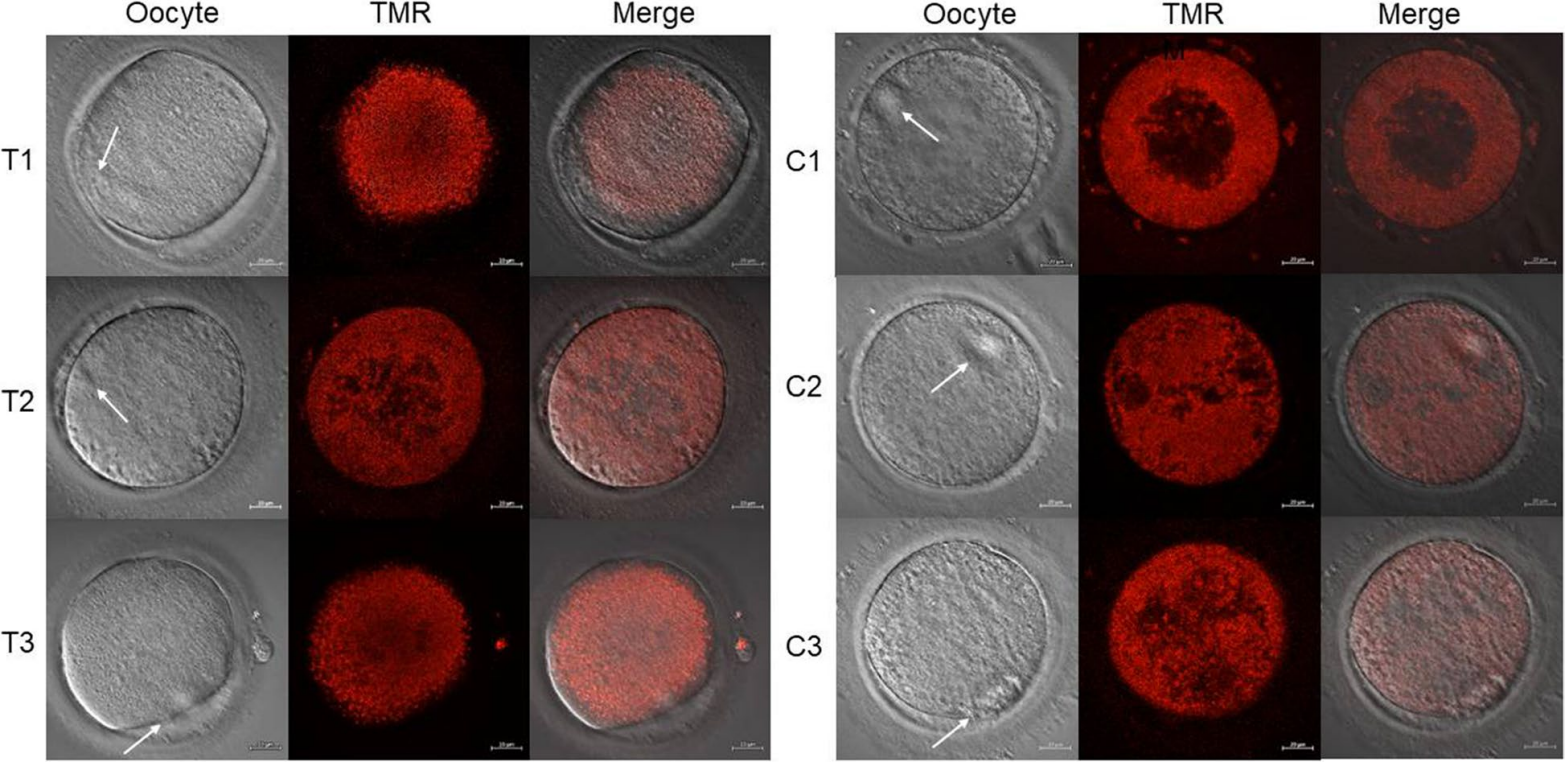
Tetramethylrhodamine methyl ester (TMRM) fluorescence was detected by confocal microscope at 525 nm to indicate mitochondria distribution of oocytes cytoplasm. (C1, C2 and C3) oocytes at less than 4 h cultivation after PB1 extrusion; (T1, T2 and T3) oocytes at 4–6 h cultivation after PB1 extrusion

The distribution of mitochondria of IVM oocytes was visualized by TMRM staining. The mitochondria in the cytoplasm of almost three IVM oocytes with less than 4 h cultivation after PB1 extrusion presented a semiperipheral distribution pattern, which indicated the location of mitochondria between the cortical region and spreading all over the cytoplasm of the oocytes (Fig. 2 C1, C2, and C3). While the mitochondria in the cytoplasm of almost three in vitro maturation oocytes with a 4–6 h’ cultivation after PB1 extrusion presented the diffused distribution pattern, indicating the state of mitochondria spreading all over the cytoplasm (Fig. 2 T1, T2, and T3).

## Discussion

Our results demonstrated that for low prognosis patients under the POSEIDON classification, immature oocytes could be utilized to provide more utilizable embryos for patients when they were performed ICSI 4–6 h after the PB1 extrusion on the oocytes retrieval day. We performed a multivariable logistic regression-GEE model to get the effect of ICSI timing on fertilization, embryonic development, and clinical results. To the best of our knowledge, it was for the first time ICSI timing was studied in a new clinical scenario-low prognosis patients under the POSEIDON classification, and a generalized estimate equation was used to avoid repeated measurement effects of participant characteristics.

IVM was first utilized successfully in 1991 [12], and afterward, it was mainly used for polycystic ovary syndrome (PCOS) patients. To avoid hyperstimulation, PCOS patients were seldom stimulated, so almost all the retrieved oocytes were immature. To get a higher efficiency for these immature oocytes, ICSI timing was implored in several studies. Hyun et al. involved PCOS patients who underwent an hCG-primed IVM cycle. They demonstrated the optimal ICSI timing was 2–4 h after the PB1 extrusion for maturation of MI oocytes, and they got a 12.7% implantation rate (19/150) [13]. Ranganath et al. also studied the optimal ICSI timing on GV oocytes for PCOS patients. They reported 5–6 h post 1 PB extrusion was the optimal ICSI timing and yielded the highest embryo utilization rate [14]. This difference in the results could be due to the different culture conditions for obtaining the mature oocytes. However, we could conclude from these two studies that ICSI timing after the PB1 extrusion may play an essential role in developing IVM embryos.

For normally stimulated IVF patients, immature oocytes also existed at retrieval, and the nature of the immature oocytes differed from that of PCOS patients. So the optimal ICSI timing after the PB1 extrusion also deserves imploring to get a higher utilization rate of the immature oocytes from normally stimulated IVF patients. Álvarez et al. studied patients containing only immature oocytes at retrieval with normal COS protocols. They reported a 2–6 h time interval between the first polar body extrusion, and ICSI performance was suggested to be superior to the 8–11 h and 23–26 h interval for IVM metaphase I oocytes. With this strategy, they got 1 live birth from 13 transferred cycles [15]. Lee et al. also studied IVF routine immature oocytes for patients with low functional ovarian reserve (LFOR) [6]. In their study, immature oocytes were checked at 12 h intervals. ICSI was performed on those oocytes that finished extruding the PB1, so there was no information about the time interval between the PB1 extrusion and ICSI performance. They reported a fertilization rate of 64.7%, and 1 in 7 patients got pregnant and delivered a healthy baby for LFOR patients only having immature oocytes. In our study, for low prognosis patients under the POSEIDON classification, we first concluded that ICSI performing on the same day of oocytes retrieval (P-ICSI and R-ICSI) was superior to ICSI performing overnight (E-ICSI), consistent with a previous study [16]. When considering the time interval between the first polar body extrusion and ICSI performance on the oocytes retrieval day, we concluded a 4–6 h interval (P-ICSI) might be superior to a less than 4 h interval (R-ICSI) with higher blastocyst formation and implantation rate though without significant difference. To the best of our knowledge, this study, for the first time, offered evidence for the effect of ICSI timing on IVM oocytes for low prognosis patients under the POSEIDON classification and got the relatively highest implantation rate for IVM oocytes.

The mechanisms under the optimal ICSI timing are still unclear nowadays. As a result, meiotic spindle formation and mitochondria distribution were studied a lot. Yu et al. reported the 2- to 3- h and 4- to 5- h duration of cultivation after PB1 extrusion were beneficial for chromosome alignment and tubulin organization for IVM oocytes, and inadequate maturation timing (0 to 1 h or exceeding 8 h) may impair the developmental efficiency and embryo quality of IVM oocytes [17]. The immature oocytes collected in their study failed to extrude PB1 after routine ICSI performing and in vitro cultivated for another 12 h. Furthermore, prolonged culture impaired developmental competence in both their and our study though investigated subjects differed.

Previous studies revealed that the redistribution of mitochondria was coordinated with oocyte maturation and that a lack of redistribution of mitochondria suggested incomplete cytoplasmic maturation and lower developmental competence [18–20]. Liu et al. concluded the distribution pattern of mitochondria was changed from peripheral in immature oocytes to diffused in mature oocytes after in vitro maturation [21]. In our study, we observed the distribution of mitochondria for in vitro maturation oocytes at less than 4 h culture after PB1 extrusion was more semiperipheral, while the distribution of mitochondria for in vitro maturation oocytes at 4–6 h culture after PB1 extrusion was more diffused. Notably, this explained the better embryonic development outcomes for P-ICSI than R-ICSI immature oocytes.

Here, we reported an efficient strategy of utilizing the immature oocytes for the low prognosis patients under the POSEIDON criteria. Though it was more inconvenient than routine treatment, it warranted performing, considering the high implantation rate of these IVM embryos. However, we could not obtain a significant difference between P-ICSI and R-ICSI groups for the limited sample size. In the future, randomized controlled trials with large sample sizes should be conducted to implore the difference between these two groups. Another shortcoming of this study was that the exact time of PB1 extrusion in the E-ICSI group was unknown. So we cannot determine the exact time interval between PB1 extrusion and ICSI performing, and this could be a co-factor relevant with the embryo developmental outcome difference. In the future, combined with the advantages of the time lapse incubator, better experiments can be designed to exclude the interference of this factor.

Conclusions: Immature oocytes were valuable embryo sources for low prognosis patients under the POSEIDON criteria, and the IVM oocytes at 4–6 h after PB1 extrusion on the oocytes retrieval day had the diffused mitochondria distribution pattern, making this the optimal ICSI timing for these IVM oocytes.

## Data Availability

The datasets used or analyzed in this study are available from the corresponding authors on reasonable request.

## Abbreviations

IVF-ET: In vitro fertilization embryo transfer
POSEIDON: Patient-Oriented Strategies Encompassing Individualized Oocyte Number
CDR: Cumulative delivery rate
MI: Metaphase I
GV: Germinal vesicle
PB1: The first polar body
IVM: In vitro maturation
ICSI: Intracytoplasmic sperm injection
MII: Metaphase II
OAT: Oligoasthenoteratozoospermia
COS: Controlled ovarian stimulation
hCG: Human chorionic gonadotropin
OCCCs: Oocyte-corona radiata-cumulus oophorus complexes
GEE: A generalized estimate equation
R-ICSI: Routine-ICSI
P-ICSI: Precise-ICSI
E-ICSI: Extented-ICSI
ANOVA: One-way analysis of variance
MGE: Morphological good embryos
TMRM: Tetramethylrhodaminemethyl ester
AMH: Anti-Müllerian hormone
FSH: Follicle stimulation hormone
